# Risk stratification in clinical trials: a subgroup to be encouraged

**DOI:** 10.1186/1745-6215-14-S1-O104

**Published:** 2013-11-29

**Authors:** Tim Clayton, Stuart Pocock

**Affiliations:** 1 London School of Hygiene & Tropical Medicine, London, UK

## 

The analysis of randomised controlled trials by subgroups of individuals (e.g. according to age or gender) remains controversial and often misinterpreted. It is recognised that such analyses should be limited to a few pre-specified key baseline factors. In addition an appropriate analysis typically calculates effect estimates and confidence intervals within such subgroups together with an overall interaction test rather than calculating separate p-values within each category of the subgroup.

However, patients vary considerably often presenting with multiple risk factors for the outcome of interest. Hence an alternative approach to analysing subgroups is proposed whereby patients are analysed according to their underlying risk rather than individual characteristics and a single interaction test performed between underlying risk and treatment. Patients can be categorised into several ordered risk groups and the absolute and relative risk reduction in each group presented. This helps the decision making process for an individual patient when considering appropriate treatment strategies. For instance, even if a beneficial relative risk is steady across groups, the absolute benefits in the low-risk group may be too small to justify the new treatment’s use.

These issues will be illustrated using data from trials in acute coronary syndrome. The FIR collaboration combined data from three trials (FRISC-II, RITA-3 and ICTUS) interventional and conservative strategies. The ACUITY trial assessed the impact of three anti-thrombotic therapies for reducing clinical outcomes and major bleeding.

We recommend that risk stratified subgroup analyses be routinely reported in trials that claim a treatment benefit.

